# Risk of joint replacement surgery in Taiwanese female adults with systemic lupus erythematosus: a population-based cohort study

**DOI:** 10.1186/s12891-019-2698-6

**Published:** 2019-07-06

**Authors:** Chien-Han Chen, Chia-Wen Hsu, Ming-Chi Lu

**Affiliations:** 1 Division of Obstetrics and Gynecology, Dalin Tzu Chi Hospital, Buddhist Tzu Chi Medical Foundation, Chiayi, Taiwan; 2 Department of Medical Research, Dalin Tzu Chi Hospital, Buddhist Tzu Chi Medical Foundation, Chiayi, Taiwan; 3 Division of Allergy, Immunology and Rheumatology, Dalin Tzu Chi Hospital, Buddhist Tzu Chi Medical Foundation, No. 2, Minsheng Road, Dalin, Chiayi, 62247 Taiwan; 4 School of Medicine, Tzu Chi University, Hualien, Taiwan

**Keywords:** Systemic lupus erythematosus, Skin and connective tissue diseases, Joint replacement, Replacement arthroplasty

## Abstract

**Background:**

Female patients with systemic lupus erythematosus (SLE) are prone to have musculoskeletal system involvement, which could lead to joint damage. However, few studies have assessed the incidence of arthroplasty in female patients with SLE. The aim of this study was to investigate the risk of total hip replacement surgery and total knee replacement surgery in patients with SLE.

**Methods:**

We identified 577 female patients with newly diagnosed SLE between 2000 and 2012 using the Taiwan’s National Health Insurance Research Database. A comparison cohort was constructed with female patients without SLE in a ratio of 5:1, based on frequency matching for 10-year age interval, and index year, for each patient with SLE. Both cohorts were followed until a diagnosis of the study outcomes or the end of the follow-up period.

**Results:**

Female patients with SLE showed a significantly higher incidence of receiving total hip replacement surgery (adjusted incidence rate ratio [aIRR] 6.47; *P* < 0.001), but not total knee replacement surgery (aIRR 1.81; *P* = 0.227). Moreover, age-group stratified analyses indicated a high incidence for receiving total hip replacement surgery among young female patients with SLE (aIRR 7.70; *P* = 0.001).

**Conclusion:**

Young female patients with SLE had a high risk of receiving total hip replacement surgery, but not total knee replacement surgery.

## Background

Systemic lupus erythematosus (SLE) is a prototype of systemic autoimmune disease predominately affected women during their childbearing age with an age of onset in late teens and early 40s [1]. Asian countries including Taiwan are at a high risk for developing SLE [2], and the incidence rate was 4.87 per 100,000 population in Taiwan during 2003–2008 [3]. SLE frequently attacks skin, kidney, and nerve, musculoskeletal, and hematological systems, which can lead to increased morbidity and mortality in patients with SLE. The prevalence, clinical manifestations, and severity of SLE vary between different ethnic groups and countries, suggesting different genetic and environmental factors could contribute to the pathogenesis of SLE [4].

Although joint involvement is usually mild and only causing pain over peripheral joints in patients with SLE, current evidence suggests that patients with SLE could have active arthritis, which might lead to joint deformities [5, 6]. In addition, Mertelsmann-Voss et al. reported an increasing risk of receiving arthroplasty over the hip and knee joints in patients with SLE during late 20th to early twenty-first century in the United States [7]. In addition, female patients with SLE are prone to have musculoskeletal involvement compared with male patients [8]. We hypothesized that female patients with SLE may have an increased risk of joint damage, leading to an increased need of arthroplasty. Therefore, the purpose of this study was to investigate the risk of total hip replacement surgery (THR) and total knee replacement surgery (TKR) in female patients with SLE using a nationwide, population-based health claims database in Taiwan.

## Methods

### Study design and data sources

This study used a nationwide, population-based, retrospective cohort design to analyze the data available from the National Health Insurance Research Database (NHIRD). The study protocol was reviewed and approved by the institutional review board of the Dalin Tzu Chi Hospital, Buddhist Tzu Chi Medical Foundation, Taiwan (No. B10104020).

### Identification of the SLE cohort and a comparison cohort

The methodology in assembling the SLE and comparison cohorts and the identification of outcome variables has been previously described [9]. Using the 2000–2012 catastrophic illness datafile, female patients with newly diagnosed SLE were defined as new and successful female applicants for the certificate of catastrophic illness with SLE (International Classification of Diseases, Ninth revision, clinical modification, ICD-9-CM code 710.0). In Taiwan, patients who suffer from a list of more than 30 categories of serious diseases, including SLE, is eligible to apply for a catastrophic illness certificates from the National Health Insurance Administration (NHIA) to receive exemption from copayments for healthcare expenses related to their diseases. The certificate is issued to patients only after their medical records and serological reports have been evaluated by the NHIA based on the 1997 American College of Rheumatology (ACR) revised criteria for the classification of SLE [10]. The index date for the SLE patients was defined as the date of the application of catastrophic illness certificate. Women who were age 20.0 to 80.0 years of age on the index date were included in the study.

The comparison cohort was constructed from the 2000 Longitudinal Health Insurance Database (LHID 2000), which contained claims records available from January 1, 2000 to December 31, 2012. The LHID 2000 is a subset of the NHIRD containing health claim data for one million beneficiaries sampled randomly, based on sex and age-group stratification from all health insurance enrollees in the Registry of Beneficiaries of the NHIRD in 2000. It represents approximately 5% of the 23.75 million Taiwanese population enrolled in 2000. To assemble the comparison cohort, five patients were selected, based on frequency matching for 10-year age interval and index year, for each patient with SLE. Patients with SLE were excluded during the selection of the comparison cohort.

### Identification of total hip replacement surgery and total knee replacement surgery

We followed both the SLE cohort and the comparison cohort to the occurrence of our study events or the end of the follow-up period. The two study events were THR and TKR. A diagnosis of THR and TKR was defined in this study as a principal inpatient ICD-9-CM code 81.51 and 81.54, respectively. Patients in the SLE and comparison cohorts receiving THR and TKR before the index date were excluded. Potential confounders, including fracture of the lower limb (ICD-9-CM codes 820–829) and obesity (ICD-9-CM code 278.0x), were also identified from the LHID 2000.

### Statistical analysis

We compared the basic characteristics between the SLE cohort and the comparison cohort using t-test or Chi-square test, as appropriate. For the SLE cohort and the comparison cohort, incidence rate per 100,000 person-years was separately calculated for THR and TKR. Incidence rate ratios (IRRs) for the outcome variables were calculated via Poisson regression models (generalized linear models with a Poisson log-linear link function and person-years as the offset variable), with and without adjusting for the potential confounding factors, including fracture of the lower limb and obesity. In addition, subgroup analyses were conducted with stratification by age groups (20–44, 45–64, and 65–80 years). All statistical tests were conducted as two-sided and a *P* value of < 0.05 was considered significant. All statistical analyses were performed using IBM SPSS Statistics for Windows, Version 24.0 (IBM Corp, Armonk, NY, USA).

## Results

There are no significant differences between female patients with SLE and comparison cohort in age, socioeconomic status, and geographic region. Patients with SLE have higher proportions of obesity (2.6% vs. 1.4%; *P* = 0.033) and fracture of the lower limb (8.1% vs. 4.4%; *P* < 0.001) compared to those in the comparison cohort (Table 1).

**Table 1:** Basic characteristics of the systemic lupus erythematosus cohort and comparison cohort (*N* = 3462)

Variable	n (%)		*P* value
	systemic lupus erythematosus cohort 577 (16.7)	comparison cohort 2885 (83.3)	
Age, years, mean (SD)	41.0 (14.7)	40.5 (12.9)	0.432
Obesity	15 (2.6)	40 (1.4)	0.033
Fracture of the lower limb	47 (8.1)	127 (4.4)	< 0.001
Socioeconomic status (*n* = 3364)			0.058
Low	212 (37.7)	914 (32.6)	
Medium	183 (32.6)	960 (34.3)	
High	167 (29.7)	928 (33.1)	
Geographic region (*n* = 3361)			0.646
Northern	352 (64.2)	1812 (64.4)	
Central	95 (17.3)	434 (15.4)	
Southern	93 (16.9)	511 (18.2)	
Eastern	9 (1.6)	55 (2.0)	

*SD* standard deviation

The incidence rates and IRRs of THR and TKR for the SLE cohort and the comparison cohort were shown in Table 2. Patients with SLE exhibited a significantly higher incidence of receiving THR compared with the comparison cohort (IRR 8.98; 95% CI: 3.53–22.80, *P <* 0.001 and adjusted IRR 6.47; 95% CI: 2.43–17.22, *P <* 0.001, respectively). No significant differences were noted between the IRRs of receiving TKR between the patients with SLE and those of the comparison cohort. Table 3 showed the incidence rates and IRRs of THR and TKR for the SLE cohort and the comparison cohort, stratified by three age groups. The IRR for THR in SLE patients with the age of 20–44 years was marked elevated (adjusted IRR 7.70; 95% CI: 2.19–27.12, *P* = 0.001) compared with those in the comparison cohort. However, the IRRs for THR were not significantly different between the SLE cohort and the comparison cohort in patients aged 45–64 and 65–80. Moreover, no patients were found to receive TKR in the 20–44 years age group in the comparison cohort and no patients were found to receive TKR in the 65–80 years in the SLE cohort. The adjusted IRRs for TKR in all age groups were not significantly different between the SLE cohort and comparison cohort.

**Table 2:** Incidence rates and incidence rate ratios of surgery for systemic lupus erythematosus cohort and comparison cohort (*N* = 3462)

Type of surgery (ICD-9-CM)	systemic lupus erythematosus cohort			comparison cohort			IRR (95% CI)	aIRR (95% CI)
							*P* value	*P* value
	No. of patients	Person-years	IR	No. of patients	Person-years	IR		
THR (81.51)	12	3643.4	329.36	7	19,080.9	36.68	8.98 (3.53–22.80) < 0.001	6.47 (2.43–17.22) < 0.001
TKR (81.54)	6	3659.9	163.94	15	19,042.0	78.77	2.08 (0.81–5.36) 0.129	1.81 (0.69–4.75) 0.227

*aIRR* adjusted incidence rate ratio, *IR* incidence rate, *THR* total hip replacement surgery, *TKR* total knee replacement surgery

aIRRs were adjusted for obesity, fracture of the lower limb, socioeconomic status, and geographic region

**Table 3:** Incidence rates and incidence rate ratios of surgery for systemic lupus erythematosus cohort and comparison cohort, with stratification by age group (N = 3462)

Type of surgery (ICD-9-CM)	Age group (years)	systemic lupus erythematosus cohort			comparison cohort			IRR (95% CI)	aIRR (95% CI)
								*P* value	*P* value
		No. of patients	Person-years	IR	No. of patients	Person-years	IR		
THR (81.51)	20–44	8	2391.0	334.6	4	12,548.0	31.9	10.53 (3.17–34.96) < 0.001	7.70 (2.19–27.12) 0.001
	45–64	3	1087.8	275.8	2	5563.3	35.9	7.67 (1.28–45.90) 0.026	4.69 (0.64–34.07) 0.127
	65–80	1	164.6	607.5	1	933.6	107.1	5.67 (0.36–90.66) 0.220	1.98 (0.10–40.49) 0.657
TKR (81.54)	20–44	1	2417.4	41.4	0	12,604.5	0.0	NC	NC
	45–64	5	1071.4	466.7	10	5532.2	180.8	2.58 (0.88–7.55) 0.083	2.66 (0.90–7.90) 0.078
	65–80	0	171.1	0.0	5	905.3	552.3	NC	NC

*aIRR* adjusted incidence rate ratio, *IR* incidence rate, *NC* not calculable, *THR* total hip replacement surgery, *TKR* total knee replacement surgery

aIRRs were adjusted for obesity, fracture of the lower limb, socioeconomic status and geographic region

## Discussion

In this secondary cohort analysis, we found that that young female patients with SLE suffered from a high incidence for receiving THR compared with the patients without SLE. Few studies have addressed the risk of THR and TKR in patients with SLE, and the incidence and incidence rate ratios compared with non-SLE patients were unclear. Mukherjee et al. reported that patients with SLE had an increased risk of receiving TKR, but not THR in a United Kingdom case-control study [11], which is different from our findings. The difference might be related to the different manifestations and severity of SLE in different ethnic groups (European versus Asian ancestry) [12].

In the young age group (20–44 years), patients with SLE exhibited more than 7-fold risk for receiving THR. Among these young patients with SLE, seven patients received THR due to osteonecrosis and one patient due to osteoarthritis. In the comparison cohort, three patients received THR due to osteoarthritis and one patient due to congenital abnormality of hip. Osteonecrosis is also known as avascular necrosis of bone, and it is the death of bone tissue related to a decreased in blood supply. Patients with osteonecrosis would suffer from the collapse of the bony structure, resulting in joint pain and loss of function. Patients with SLE are well known to have an increased risk of osteonecrosis [13]. Our study showed a large increased risk of THR, particularly in young SLE patients. This finding is consistent with a previous report in Korean patients with SLE [14]. The occurrence of osteonecrosis in patients with SLE could be affected by various factors. The long-term use of corticosteroid is certainly a strong risk factor of osteonecrosis, but neuropsychiatric manifestations of SLE, vasculitis, hypertension, serositis, and renal disease could also increase the risk of osteonecrosis [15]. In addition, it is estimated that more than half of the SLE patients who have osteonecrosis will progress and finally require surgical intervention [16]. The final treatment for severe osteonecrosis is a joint replacement surgery of the respective joints, which is an economic burden for both the healthcare system and the patients. Furthermore, patients with SLE receiving THR may suffer from a higher risk of surgical complications, such as deep vein thrombosis, acute kidney injury, and wound infections [17]. Therefore, the judicious use of corticosteroid with the addition of immunosuppressive agents is suggested to lower the risk of osteonecrosis. Early recognition and timely treatment for osteonecrosis is also important. Another potential cause of THR in patients with SLE is femoral neck fractures secondary to osteoporosis. However, we did not observe this cause of THR in the present study. Osteoporosis generally occurs in senile people, but the age of our SLE cohort is younger, which could explain the absence of THR due to femoral neck facture attributed to osteoporosis.

Among the six patients with SLE who received TKR in this study, one patient was due to osteonecrosis and the remaining five patients were due to osteoarthritis. Osteoarthritis tends to develop in elderly patients, and since the SLE patients in this study were relatively young, they were less likely to receive TKR because of osteoarthritis.

Previous research indicated that obesity was a risk factor for receiving TKR and THR [18, 19], and patients with SLE can have a higher prevalence of obesity as a result of their long-term use of steroids. It should be noted that the identification of obesity in this study was based on the ICD-9-CM code 278.0x, which represents patients with morbid obesity. The prevalence of morbid obesity varies from 0.88% in Spain [20], 5% in UK [21], and 6% in USA [22]. The prevalence of morbid obesity in our comparison cohort was 1.4%, which is similar to that in Korea 0.9% [23].

A few limitations in this study should be noted. First, due to the restriction of the NHIRD, serological results for SLE and imaging reports for knee and hip joint were unavailable. Second, the diagnosis of SLE was based on catastrophic illness certificate. Nevertheless, the issue of the certificate was randomly audited by the NHIA to ensure their accuracy. Third, the risks of THR and TKR in male patients with SLE were not assessed due to the small sample size of male patients with SLE and milder degree of musculoskeletal system involvement in male patients with SLE [8]. Fourth, the data used in this study were retrospective in nature, and the need of prospective ad hoc studies will be required to overcome some of the aforementioned limitations.

## Conclusions

In conclusion, this secondary cohort analysis of a nationwide, population-based health claims database showed that female patients with SLE had a higher risk of receiving THR, but not TKR. A high risk of receiving THR among young female SLE patients requires attention.

## Data Availability

The data that support the findings of this study are available on request from the corresponding author. The data are not publicly available due to the Taiwan Personal Information Protection Act.

## Abbreviations

ACR: American College of Rheumatology
aIRR: Adjusted incidence rate ratio
ICD-9-CM: International Classification of Diseases, 9th revision, Clinical Modification Declarations
IRR: Incidence rate ratios
LHID: Longitudinal Health Insurance Database
NHIA: National Health Insurance Administration
NHIRD: National Health Insurance Research Database
SLE: Systemic lupus erythematosus
THR: Total hip replacement surgery
TKR: Total knee replacement surgery
